# Newcastle disease virus infection induces parthanatos in tumor cells via calcium waves

**DOI:** 10.1371/journal.ppat.1012737

**Published:** 2024-12-02

**Authors:** Yang Qu, Siyuan Wang, Hui Jiang, Ying Liao, Xusheng Qiu, Lei Tan, Cuiping Song, Venugopal Nair, Zengqi Yang, Yingjie Sun, Chan Ding

**Affiliations:** 1 Department of Avian Infectious Diseases, Shanghai Veterinary Research Institute, Chinese Academy of Agricultural Science, Shanghai, P. R. China; 2 School of Agriculture and Biology, Shanghai Jiao Tong University, Shanghai, P. R. China; 3 College of Veterinary Medicine, Northwest A&F University, Yangling, Shaanxi, P. R. China; 4 College of Veterinary Medicine, South China Agricultural University, Guangzhou, P.R. China; 5 Avian Oncogenic viruses group, UK-China Centre of Excellence on Avian Disease Research, The Pirbright Institute, Pirbright, Guildford, Surrey, United Kingdom; Icahn School of Medicine at Mount Sinai, UNITED STATES OF AMERICA

## Abstract

Parthanatos is distinct from caspase-dependent apoptosis in that it does not necessitate the activation of caspase cascades; Instead, it relies on the translocation of Apoptosis-inducing Factor (AIF) from the mitochondria to the nucleus, resulting in nuclear DNA fragmentation. Newcastle Disease Virus (NDV) is an oncolytic virus that selectively targets and kills tumor cells by inducing cell apoptosis. It has been reported that NDV triggers classic apoptosis through the mitochondrial pathway. In this study, we observed that NDV infection induced endoplasmic reticulum stress (ERS), which caused a rapid release of endogenous calcium ions (Ca^2+^). This cascade of events resulted in mitochondrial depolarization, loss of mitochondrial membrane potential, and structural remodeling of the mitochondria. The overload of Ca^2+^ also initiated an increase in mitochondrial membrane permeability, facilitating the transfer of AIF to the nucleus to induce apoptosis. Damaged mitochondria produced excessive reactive oxygen species (ROS), which further exacerbated mitochondrial damage and increased mitochondrial membrane permeability, thus promoting additional intracellular Ca^2+^ accumulation and ultimately triggering an ROS burst. Collectively, these findings indicate that NDV infection promotes excessive calcium accumulation and ROS generation, leading to mitochondrial damage that releases more calcium and ROS, creating a feedback loop that exacerbates AIF-dependent parthanatos. This study not only provides a novel perspective on the oncolytic mechanism of NDV but also highlights new targets for antiviral research.

## Author summary

Apoptosis serves as a cellular self-defense mechanism against viral infections, while also being a consequence of viral proliferation within infected cells. Mitochondria play a critical role in amplifying the stress associated with viral infections, as they sustain damage in response to intracellular apoptosis-inducing signals. Our observations indicate that NDV infection leads to Ca^2+^ accumulation and excessive ROS production, which in turn results in mitochondrial depolarization, loss of membrane potential, increased permeability, and the subsequent release and transport of AIF to the nucleus, ultimately triggering tumor cell death. These findings provide compelling evidence for an NDV-induced parthanatos mechanism and underscore the significant roles of calcium and ROS in mitochondrial apoptotic pathways.

## Introduction

Apoptosis is a process of programmed cell death that is tightly regulated by a series of complex pathways and is considered a suicidal act to eliminate unwanted and potentially dangerous cells [1,2]. This process is crucial for the normal development and homeostasis of multicellular organisms and plays a significant role in the pathogenesis of various viral infections [3,4]. Apoptosis can be initiated through either the extrinsic pathway mediated by cell death receptor or the intrinsic pathway, which is associated with mitochondrial dysfunction [5]. Caspases, a family of cysteine-dependent aspartate-directed proteinases, are central to the apoptosis process. They are activated by both intrinsic and extrinsic apoptotic signals, with Caspase-9 and Caspase-8 serving as initiators of the intrinsic and extrinsic apoptotic pathways, respectively, while Caspase-6 and Caspase-3/7 function as effectors [6,7]. Mitochondria serve as distribution hubs for apoptotic activators, containing various proteins associated with apoptosis within their membrane space. For example, cytochrome C (Cyt C) is involved in caspase-dependent mitochondrial apoptosis [8]. Additionally, molecules such as AIF and endonuclease G (Endo G) are released from mitochondria and induce DNA damage and fragmentation independently of caspases, thus playing a regulatory role in apoptosis [9,10].

AIF is a mitochondrial flavoprotein located in the mitochondrial intermembrane space, where it is essential for maintaining mitochondrial morphology and cristae structure [11,12]. The activation of the DNA repair enzyme poly(ADP-ribose) polymerase-1 (PARP-1) has been shown to lead to elevated levels of poly(ADP-ribose) (PAR) polymers, which subsequently promote the release of AIF[13]. AIF recruits macrophage migration inhibitory factor (MIF) to the nucleus, where it cleaves genomic DNA into high molecular weight fragments (~50 kb), ultimately resulting in cell death. This caspase-independent cell death program is termed parthanatos [14–16]. Furthermore, additional studies have shown that calcium imbalance regulates AIF release by activating the calcium-dependent cysteine protease calpain [17,18]. AIF-dependent parthanatos exists in many diseases, including ischemia-reperfusion injury, inflammatory injury, ROS-induced injury, and neurobiological diseases [19–21]. In recent years, numerous viruses belonging to different families have been demonstrated to induce AIF-dependent parthanatos during their infection cycle [22–25].

A key event in apoptosis is mitochondrial outer membrane permeabilization (MOMP), which leads to the release of pro-apoptotic factors. For instance, the Bcl-2 family proteins Bax and Bak oligomerize to form pores in the mitochondrial outer membrane (MOM), thereby facilitating programmed cell death [26–28]. The mitochondrial membrane permeability transition pore (mPTP) is a protein complex that connects the cytoplasm, the intermembrane space of mitochondria, and the mitochondrial matrix, which can also be used as the release channel for apoptotic molecules from the mitochondria [26,29]. In addition, several stressors, including viral proteins, toxic chemicals, ROS, and calcium signaling, directly or indirectly target mitochondria, promoting increased membrane permeability. Mitochondria play a critical role as calcium buffers; however, an overload of Ca^2+^ leads to mitochondrial stress damage and the transition of the mitochondrial membrane permeability. These changes result in the release of various apoptosis-related factors. Viral infections can enhance both extracellular Ca^2+^ influx and intracellular Ca^2+^ release, which accelerates the rise in mitochondrial Ca^2+^ levels. Mitochondrial calcium overload further exacerbates the production of ROS. In turn, excessive ROS negatively impacts the mitochondrial membrane, resulting in the increased permeability and the subsequent release of apoptotic signals [30,31]. Additionally, there are reports indicating that elevated levels of ROS are directly implicated in the AIF-dependent parthanatos process activated by PARP-1 [32,33]. However, the role of calcium and ROS as apoptotic agonists in virus-induced AIF-dependent apoptotic pathways remains unclear, and the relationship between calcium and ROS is yet to be fully elucidated.

NDV is a highly contagious and widespread pathogen among avian species, classified within the family Paramyxoviridae [34]. Additionally, NDV functions as an oncolytic virus, capable of selectively infecting human cancer tissues, replicating and proliferating within tumor cells, and directly inducing cancer cell death [35,36]. While numerous studies have investigated the mechanisms underlying of NDV-induced tumor cell death, including the exogenous death ligand pathway, the caspase-dependent apoptotic pathway, and ferroptosis [37–39], the processes of mitochondrial apoptotic pathway remain inadequately understood. Furthermore, different virulent strains of NDV induce distinct apoptosis signaling pathways and variations in immune responses, which consequently lead to differences in the mechanisms of tumor cell apoptosis induction [40]. Our previous reports demonstrated that NDV infection disrupts mitochondrial homeostasis [41], leading us to hypothesize that NDV may induce an intrinsic apoptotic pathway mediated by mitochondrial dysfunction, particularly given that mitochondria serve as crucial organelles that sense and amplify stress responses. In this study, we examined how mitochondria, as targets for calcium and ROS, respond to Ca^2+^ accumulation and ROS overproduction triggered by NDV infection, ultimately leading to AIF-dependent parthanatos. This study is a novel investigation into the oncolytic mechanism of NDV, contributing to the theoretical framework surrounding the role of oncolytic viruses in tumor cell death. It aims to provide a comprehensive understanding of the involvement of calcium signaling and reactive oxygen species in the regulation of host cell death within viral infectious disease models.

## Results

### NDV infection induces caspase-dependent apoptosis

**Fig 1:**
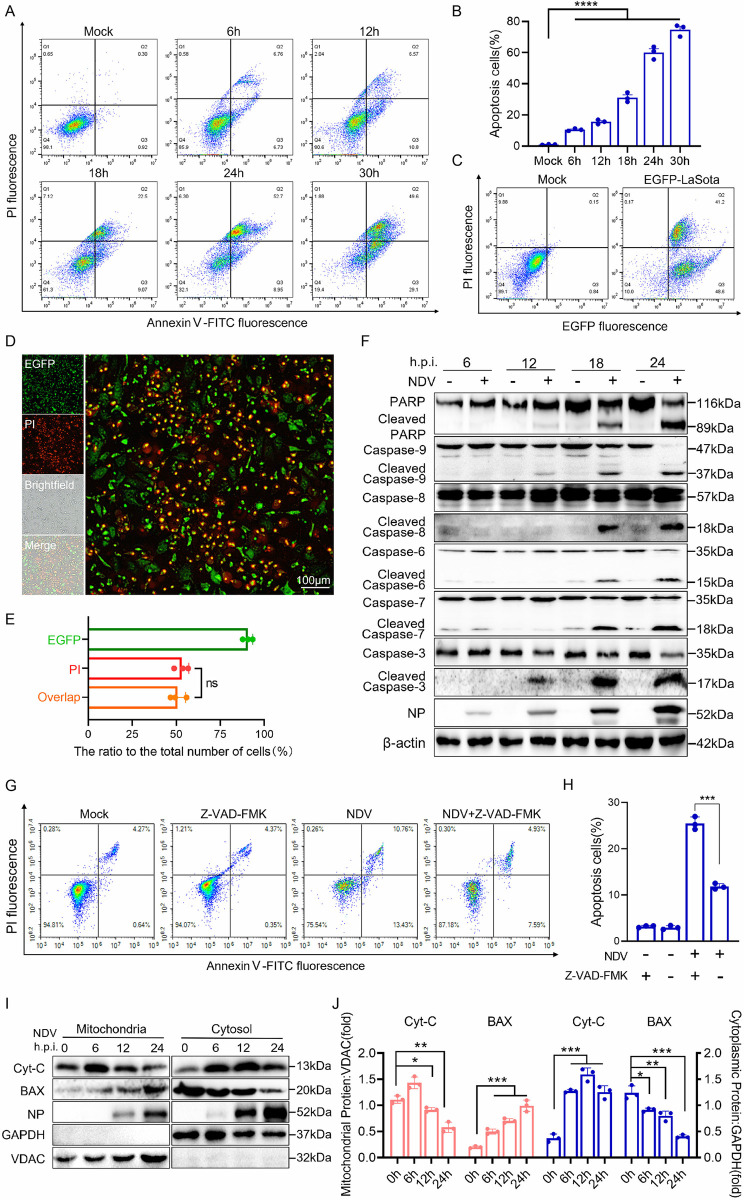
NDV infection induces caspase-dependent apoptosis. (A) Apoptosis was evaluated using flow cytometry at 6, 12, 18, 24 and 30 h post-infection with a multiplicity of infection (MOI) of 1 for NDV or a mock infection. (B) The apoptosis rates of cells at different times of infection. (C-E) HeLa cells were infected with the NDV EGFP-LaSota strain at a MOI of 5 for a duration of 24 hours, and then labeled with PI, detection of apoptotic cells by flow cytometry(C); Observation by fluorescence microscope(D); Quantification of overlapping red and green fluorescence (E). (F) PARP, Caspase-9, Caspase-8, Caspase-6, Caspase-7 and Caspase-3 protein levels were determined by western blot using β-actin as the loading control and nucleoprotein (NP) as a marker for virus infection. (G) HeLa cells were treated with or without Z-VAD-FMK (10 μM), apoptosis was detected by flow cytometry at 18 h post NDV infection or mock infection. (H) The proportion of apoptotic cells in different treatments. (I) Quantities of Cyt C and Bax proteins amount in cytosol and mitochondria were determined by western blot using glyceraldehyde 3-phosphate dehydrogenase (GAPDH) and voltage-dependent anion channel (VDAC) protein as the loading control for the cytosol and mitochondria, respectively. (J) Quantification of Cyt C and BAX expression in cytosol and mitochondria. Each bar represents the mean ± standard deviation; *P < 0.05, **P < 0.01, ***P < 0.001 ****P < 0.0001, and ns, not significant.

Numerous studies have established that the pathogenesis and oncolytic activity of NDV are associated with cell death [35,42,43]. To investigate the oncolytic properties of NDV, tumor cells including HeLa, H1299, A549, HT-29, HepG2, Huh7 and MCF7 cells were infected with Herts/33, a virulent strain of NDV, followed by an analysis of apoptosis in cells. As expected, Annexin V and propidium iodide (PI) staining confirmed that the level of apoptosis in HeLa cells increased significantly with the prolonged duration of NDV infection (Fig 1A and 1B), as well as in H1299, A549, HT-29, HepG2, Huh7 and MCF7 cells (S1A Fig). This result was further corroborated by infection experiments using the moderately virulent strain Mukteswar and the attenuated strain LaSota of NDV (S1B Fig). These results confirmed that NDV-induced apoptosis is common in tumor cells (S1A and S1B Fig), with the most pronounced effect observed in Herts/33-infected HeLa cells, consistent with our previous study [43]. Thus, the virulent Herts/33 strain and HeLa cells were chosen for subsequent experiments. In the attenuated NDV LaSota infection model expressing green fluorescent protein (EGFP-LaSota), it was observed that cells exhibiting positive PI staining also displayed green fluorescence (Fig 1C). Furthermore, fluorescence microscopy directly observed that apoptosis specifically occurred in the infected cells (Fig 1D and 1E). Correspondingly, the cleavage bands of Caspase-9, -8, -6, -7 and -3 proteins exhibited a significant increase in a time-dependent manner. PARP, a major substrate for effector caspase, was also cleaved into smaller fragments (Fig 1F). In addition, the activities of Caspase-3/7 and Caspase-6, which are effector molecules of the Caspase protein family, also increased significantly following NDV infection (S1C Fig). Z-VAD-FMK, the pan caspase inhibitor, significantly inhibited NDV-induced apoptosis (Fig 1G and 1H). Overall, these results suggested that NDV infection activates the caspase cascade, initiating both endogenous and exogenous apoptotic pathways.

For further in-depth confirmation of the intrinsic apoptosis triggered by NDV infection, we analyzed the relocalization of Bax and Cyt C upon NDV infection, which promoted MOMP and subsequently lead to the activation of Caspase-9. Western blot results revealed that upon NDV infection, the expression level of Cyt C was decreased in mitochondria, and increased in cytosol. In contrast, the expression level of Bax was increased in mitochondria, and decreased in cytosol (Fig 1I and 1J). These results were also confirmed by immunofluorescence confocal microscopy, suggesting that NDV induced the translocation of Cyt C from mitochondria to cytoplasm (S1D Fig) and Bax from cytoplasm to mitochondria (S1E Fig), respectively. Taken together, these results indicated that NDV infection facilitated Bax-induced permeabilization of the mitochondrial membrane and subsequent release of Cyt C, to activate the intrinsic apoptosis.

### NDV infection induces AIF-dependent apoptosis

**Fig 2:**
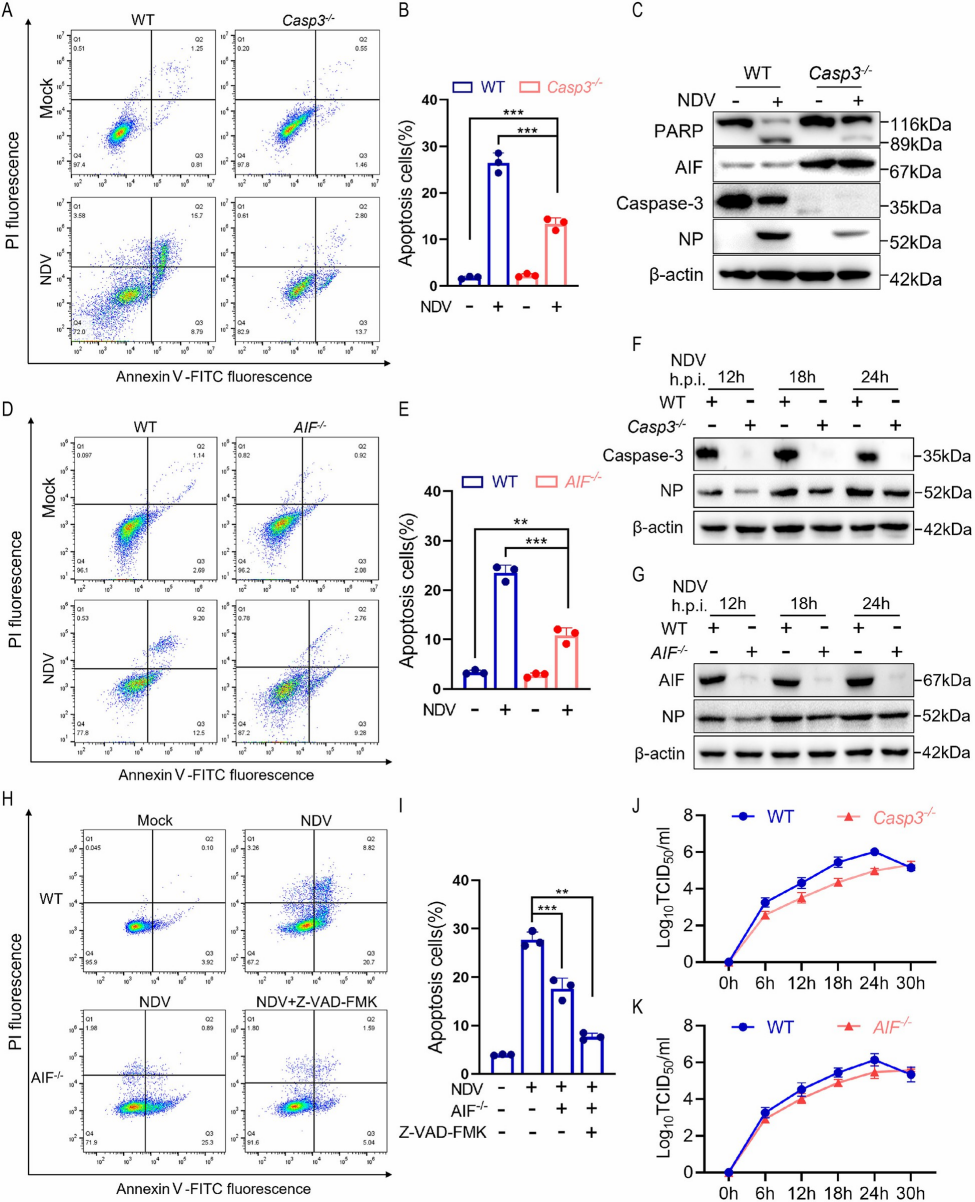
NDV infection induces AIF-dependent parthanatos. (A) Apoptosis was detected by flow cytometry at 18 h post-infection with an MOI of 1 for NDV or a mock infection of WT and *Casp3*^*-/-*^ cells. (B) The proportion of apoptotic cells in different groups. (C) Western blot analyses of the levels of PARP, AIF, and caspase-3 at 18 h post-infection with an MOI of 1 for NDV or mock infection. β-actin was used as the loading control and NP as the marker for virus infection. (D) Apoptosis was detected by flow cytometry at 18 h post-infection with a MOI of 1 for NDV or a mock infection of WT and *AIF*^*-/-*^ cells. (E) The proportion of apoptotic cells in different groups. (F and G) WT, *Casp3*^*-/-*^ or *AIF*^*-/-*^ cells were infected with NDV at an MOI of 1. Cells were harvested at 12, 18, and 24 h post-infection and protein detected NP expression levels. (H) *AIF*^*-/-*^ cells were treated with or without Z-VAD-FMK (10 μM), apoptosis was detected by flow cytometry at 18 h post-infection with an MOI of 1 for NDV. (I) The proportion of apoptotic cells in different groups. (J and K) WT, *Casp3*^*-/-*^ or *AIF*^*-/-*^ cells were infected with NDV at an MOI of 1, cell culture supernatants were subjected to the viral titer assay. The data are from three separate experiments. Each bar represents the mean ± standard deviation; **P < 0.01, ***P < 0.001.

Although Z-VAD-FMK, an efficient pan-caspase inhibitor, was unable to completely prevent NDV-induced apoptosis (Fig 1G and 1H), we hypothesized that NDV may also trigger caspase-independent apoptosis. To investigate this hypothesis, we established Caspase-3 knockout (*Casp3*^*-/-*^) cells and assessed their response to apoptosis induced by NDV infection. As expected, the knockout of caspase-3 significantly reduced, but did not entirely eliminate, the apoptosis resulting from NDV infection (Fig 2A and 2B). Interestingly, in NDV-infected *Casp3*^*-/-*^ cells, the cleavage of PARP was inhibited, while the total expression levels of AIF were found to be upregulated (Fig 2C). This observation indicates that AIF may be compensatorily activated in *Casp3*^*-/-*^ cells. These findings were consistent in the high infective dose model (S2A and S2B Fig), highlighting the key role of caspase-3 in NDV-induced apoptosis. Notably, knockout of AIF effectively also inhibited NDV-induced apoptosis (Fig 2D and 2E). This phenomenon was also observed in *Casp3*^*-/-*^ cells. Compared with virus-infected wild-type (WT) cells, the simultaneous knockout of Caspase-3 and knockdown of AIF resulted in a significant reduction in the number of apoptotic cells (S2C and S2D Fig). Similarly, treatment with the Caspase inhibitor Z-VAD-FMK in *AIF*^*-/-*^ cells significantly reduced the number of apoptotic cells induced by NDV (Fig 2H and 2I). Collectively, these data indicated that NDV infection induced caspase-dependent apoptosis and caspase-independent apoptosis involving AIF.

Notably, the knockout of Caspase-3 inhibited the expression of NDV nucleoprotein (NP) (Fig 2C). To investigate the role of apoptosis in NDV proliferation, we monitored virus replication in *Casp3*^*-/-*^ cells and *AIF*^*-/-*^ cells, respectively. The results demonstrated that the expression of the viral NP was down-regulated in both *Casp3*^*-/-*^ and *AIF*^*-/-*^ cells (Fig 2F and 2G). Examination of extracellular virus titer showed that the knockout of Caspase-3 and AIF markedly reduced the production of progeny viruses (Fig 2J and 2K). These findings suggest that apoptosis promotes viral proliferation, probably by facilitating viral release and spread.

### NDV infection activates PARP and induces mitochondrial membrane permeabilization to promote AIF nuclear translocation

**Fig 3:**
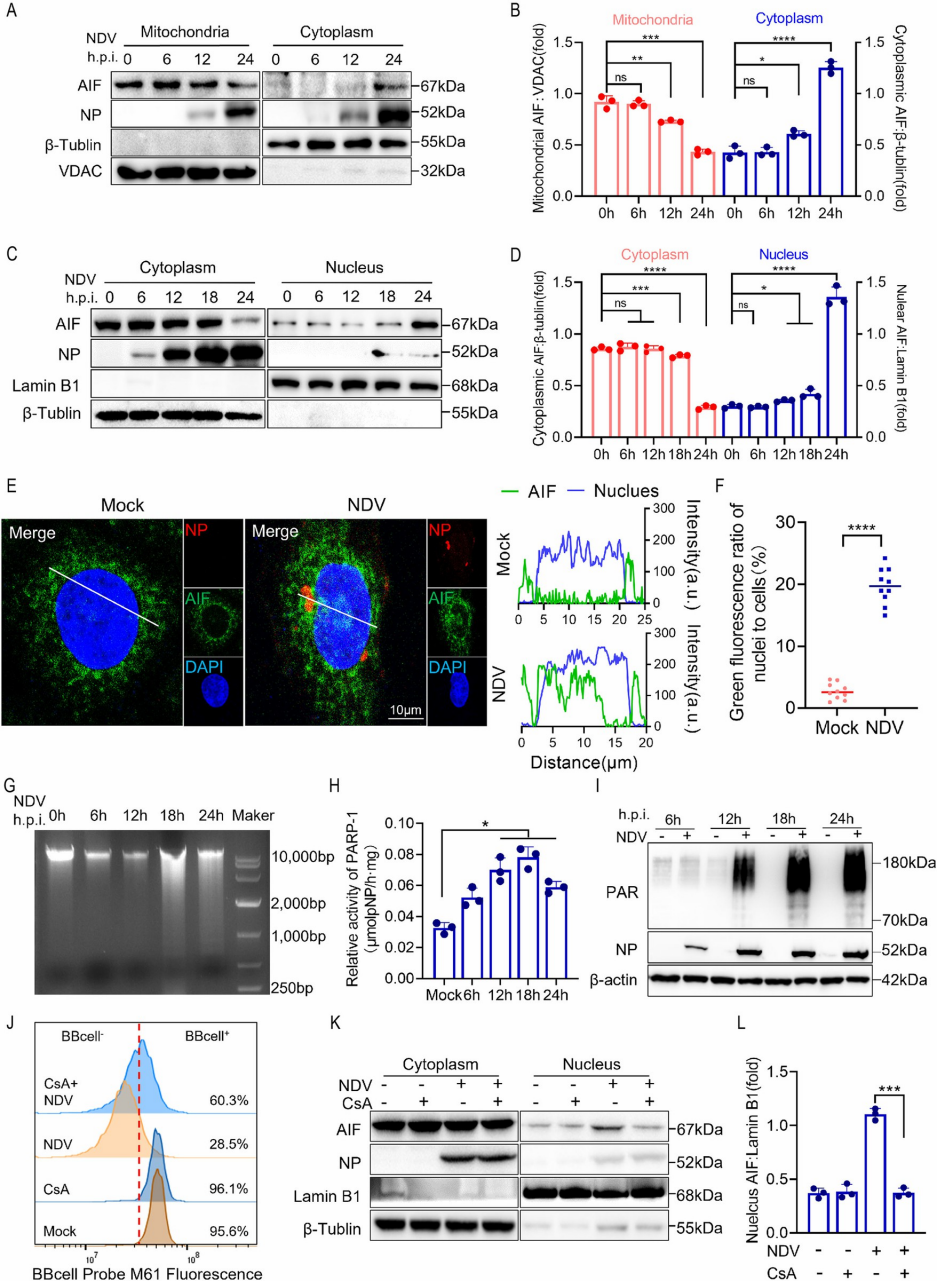
NDV infection activates PARP to induce AIF nuclear translocation. (A) Western blot analyses of the levels of AIF in cytosol and mitochondria. VDAC was used as loading control for mitochondria, β-tublin as loading control of cytosol, and NP as a marker for virus infection. (B) Quantification of AIF in mitochondria and cytosol. (C) Western blot analyses of the AIF amount in cytosol and nucleus. Lamin B1 was used as loading control for nucleus and β-tublin for cytosol. (D) Quantification of AIF in cytosol and nucleus. (E) HeLa Cells were mock treated or infected with NDV. Immunostaining was performed post-infection at 18 h post infection. Red: NDV-NP; Green: AIF with antibody; Blue: cell nucleus with DAPI. Scale bars: 10 μm. Statistical co-location analysis data are shown on the right. (F) Ratio of the intensity of green fluorescence in the nucleus. (G-I) HeLa Cells were mock treated or infected with NDV at 6, 12, 18, and 24 h, agarose gel electrophoresis was used to detect DNA integrity (G); PARP activity was determined by spectrophotometric (H); Western blot analyses of the levels of PAR formation (I). (J) HeLa cells were treated with or without CsA (10 μM), the degree of mPTP opening was detected by flow cytometry analysis of cells infected with NDV. The peak shift to the left indicates mPTP opening. (H) HeLa cells were treated with or without CsA (10 μM). Western blot analyses of the levels of AIF in cytosol and nucleus of cells at 18 h post NDV infection or mock infection. Lamin B1 and β-tublin were used as loading control for cell nuclei and cytosol respectively. (I) Quantification of AIF in mitochondria and cytosol. The MOI of NDV was set at 1 in all the experiments mentioned above. Each bar represents the mean ± standard deviation; *P < 0.05, **P < 0.01, ***P < 0.001, ****P < 0.0001 and ns, not significant.

AIF is an apoptosis inducer localized in the mitochondria. Its translocation to the nucleus is crucial for initiating the apoptotic process [15,27,44]. A significant reduction of AIF protein level in mitochondria was observed following NDV infection, accompanied by the increase of AIF protein level in the cytoplasm (Fig 3A and 3B). Subsequently, we quantified AIF expression in the nucleus. NDV infection resulted in an increase in AIF expression in nuclear extracts while simultaneously decreasing its expression in the cytoplasm (Fig 3C and 3D). Notably, the nuclear translocation of AIF induced by NDV infection was more pronounced in *casp3*^*-/-*^ cells (S3A and S3B Fig). An immunofluorescence assay was performed to further confirm the translocation of AIF following NDV infection. The analysis of AIF localization, along with the quantification of green fluorescence within the nucleus, indicated that NDV infection induces the translocation of AIF from the cytoplasm to the nucleus. (Fig 3E and 3F). In AIF-dependent parthanatos, PARP activation occurs preferentially and is accompanied by large amounts of PAR polymers formation [15,45]. Undoubtedly, NDV infection induces DNA fragmentation (Fig 3G), which triggers overactivation of PARP, as evidenced by detection of PARP activity (Fig 3H). Additionally, NDV infection notably increased the accumulation of PAR polymers after 12 h (Fig 3I). Correspondingly, the cell death induced by NDV and the formation of PAR were inhibited by the PARP-1 inhibitor 3AB (S3C–S3E Fig). In summary, NDV infection can induce the activation of PARP-1, leading to an increase in PAR polymers formation, which subsequently promotes the nuclear translocation of AIF and the process of apoptosis.

It has been reported that the release of AIF necessitates the activation of the mitochondrial pore-forming protein Bax [46], resulting in increased permeability of the mitochondrial membrane. However, it is noteworthy that the knockdown of Bax did not entirely prevent the nuclear translocation of AIF in the NDV infection model (S3F and S3G Fig). Next, we focused on the role of mPTP in NDV-induced nuclear translocation of AIF. We assessed the impact of NDV infection on mPTP activity through flow cytometry, employing the BBcellProbe M61 probe. This polar fluorescent compound readily penetrates living cell membranes and is cleaved by intracellular esterases into a membrane-impermeable form that is retained within the cells. Upon the addition of the quencher, the fluorescence emitted from the cytoplasm is quenched, enabling the detection of fluorescence exclusively from within the mitochondria. As anticipated, NDV infection significantly enhanced the opening of the mPTP (S4A and S4B Fig). Notably, treatment with the mPTP opening inhibitor cyclosporine A (CsA) effectively inhibited both NDV infection-induced mPTP opening (Fig 3J) and the subsequent nuclear translocation of AIF (Fig 3K and 3L). Given that CsA is an immunosuppressant, we investigated its impact on viral replication. The results indicated that CsA treatment can effectively inhibit viral replication (S5A and S5B Fig). These findings suggest that NDV infection facilitates mPTP opening, leading to the release of AIF. Next, we assessed whether NDV infection can cause mitochondrial dysfunction. Consistent with previous findings, NDV infection resulted in cellular mitochondrial swelling and indistinct boundaries (S4C Fig). Furthermore, extensive mitochondrial fragmentation was observed during the late stages of viral infection (S4D Fig). Western blot analyses revealed that NDV infection led to the downregulation of MFN1 and MFN2, as well as the cleavage of long OPA1 into its shorter form, thereby facilitating mitochondrial fission (S4E and S4F Fig). Additionally, NDV infection inhibited ATP production (S4G Fig) and promoted a loss of MMP (S4H and S4I Fig). In summary, NDV infection induces both structural damage and functional impairment of mitochondria, which is a prerequisite for the release of AIF from these organelles.

### NDV induces the connection between ER and mitochondria, and causes overload of intracellular and mitochondrial Ca^2+^

**Fig 4:**
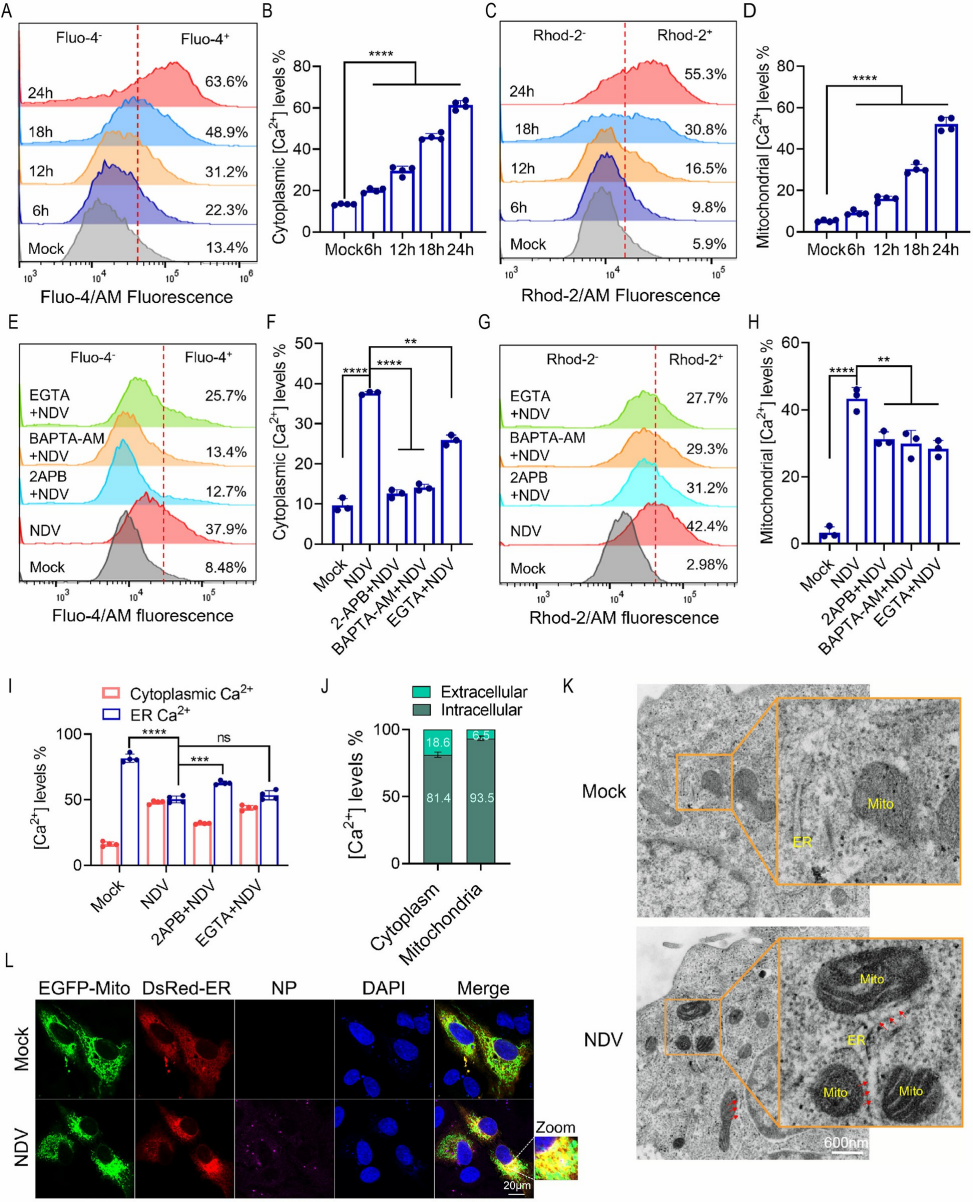
NDV infection induces ER Ca^2+^ release, cytoplasmic Ca^2+^ accumulation, and mitochondrial Ca^2+^ overload. (A-D) Cytoplasmic Ca^2+^ and mitochondria Ca^2+^ were detected by flow cytometry on mock treated cells and NDV infected cells. Fluo-4/AM was used to label cytoplasmic Ca^2+^ (A and B) and Rhod-2/AM was used to label the mitochondria Ca^2+^ (C and D). (E-H) HeLa cells were mock treated, or treated with 2-APB (100 μM), BAPTA-AM (20 μM), or EGTA (10 mM) followed by NDV infection for 18 h. Cytoplasmic Ca^2+^ (E and F) and mitochondria Ca^2+^ (G and H) were detected by flow cytometry. (I) Determination of the Ca^2+^ level. ER calcium was determined by cells stimulated with ionomycin (10 μmol/L) for 1 h before loading with the fluorescent calcium indicator Fluo-4/AM. (J) Quantification of the sources of Ca^2+^ in cytoplasm and mitochondria. (K) Electron microscopy observation. Cells were mock treated or infected with NDV and images were captured at 18 h post infection. Red arrows indicate the membrane contact between ER and mitochondria. (L) Confocal microscopy images of ER and mitochondria co-localization. Cells were mock treated, or co-transfected with pEGFP-mito and pDsRed-ER. Mitochondria (green), ER (red), NDV-NP with antibody (purple), and cell nucleus with DAPI (blue) were labeled respectively. Scale bars: 20 μm. The MOI of NDV was set at 1 in all the experiments mentioned above.Each bar represents the mean ± standard deviation; **P < 0.01, ***P < 0.001, ****P < 0.0001 and ns, not significant.

Mitochondria serve as a critical buffer for Ca^2+^ signaling, playing a pivotal role in triggering cell death during instances of Ca^2+^ accumulation and overload. Viral infections are known to almost invariably result in unstable Ca^2+^ signaling [47,48]. In light of these observations, we sought to investigate whether NDV infection induces pathological Ca^2+^ accumulation that leads to mitochondrial damage. To assess the alterations in cytoplasmic and mitochondrial calcium levels following NDV infection, we utilized Fluo-4 AM to label cytoplasmic calcium and Rhod-2 AM for mitochondrial calcium. Our findings indicate that NDV infection significantly elevated both cytoplasmic and mitochondrial Ca^2+^ levels (Fig 4A–4D). Treatment with 2-aminoethoxydiphenyl borate (2-APB), which inhibits D-myo-inositol 1,4,5-trisphosphate receptors (IP_3_R) to prevent the ER release of calcium, the intracellular calcium chelator 1,2-bis(2-aminophenoxy)ethane-N,N,N′,N′-tetraacetic acid tetrakis(acetoxymethyl ester) (BAPTA-AM), or the extracellular calcium chelator ethylene glycol tetraacetic acid (EGTA) could partially reverse the NDV-induced increase in cytoplasmic and mitochondrial Ca^2+^ (Fig 4E–4H). Notably, 2-APB exhibited a significantly greater inhibitory effect on the NDV-induced intracellular Ca^2+^ increase compared with the other two inhibitors, particularly in its ability to inhibit mitochondrial calcium accumulation. To further investigate the source of Ca^2+^, we measured calcium levels in both the cytosol and ER [49]. Infection with NDV resulted in a rapid increase in cytoplasmic Ca^2+^, which was accompanied by a decrease in ER Ca^2+^ levels. Treatment with 2-APB, but not with EGTA, facilitated the release of Ca^2+^ from the ER into the cytoplasm (Fig 4I). These findings suggest that the Ca^2+^ released from the ER, rather than the influx of extracellular Ca^2+^, plays a more significant role in response to NDV infection. Further analysis of Ca^2+^ measurements indicated that over 80% of the cytoplasmic Ca^2+^ induced by NDV infection originated from the release of intracellular Ca2+ stores, while approximately 90% of the mitochondrial Ca^2+^ was also derived from intracellular sources (Fig 4J). These results indicate that the ER serves as a crucial intracellular Ca^2+^ reservoir, contributing significantly to the increases in both intracellular and mitochondrial Ca^2+^ levels induced by NDV infection. Subsequently, we examined whether NDV infection facilitated spatial connectivity between the ER and mitochondria for calcium transport. As anticipated, NDV infection enhanced membrane contact and colocalization between the ER and mitochondria, as demonstrated by transmission electron microscopy (TEM) and confocal microscopy (Fig 4K and 4L). These findings indicate that NDV infection promotes the establishment of a connection between the ER and mitochondria, thereby creating a pathway for calcium transport.

### Inhibition of Ca^2+^ release from the ER prevents AIF-dependent parthanatos by mitigating mitochondrial dysfunction

**Fig 5:**
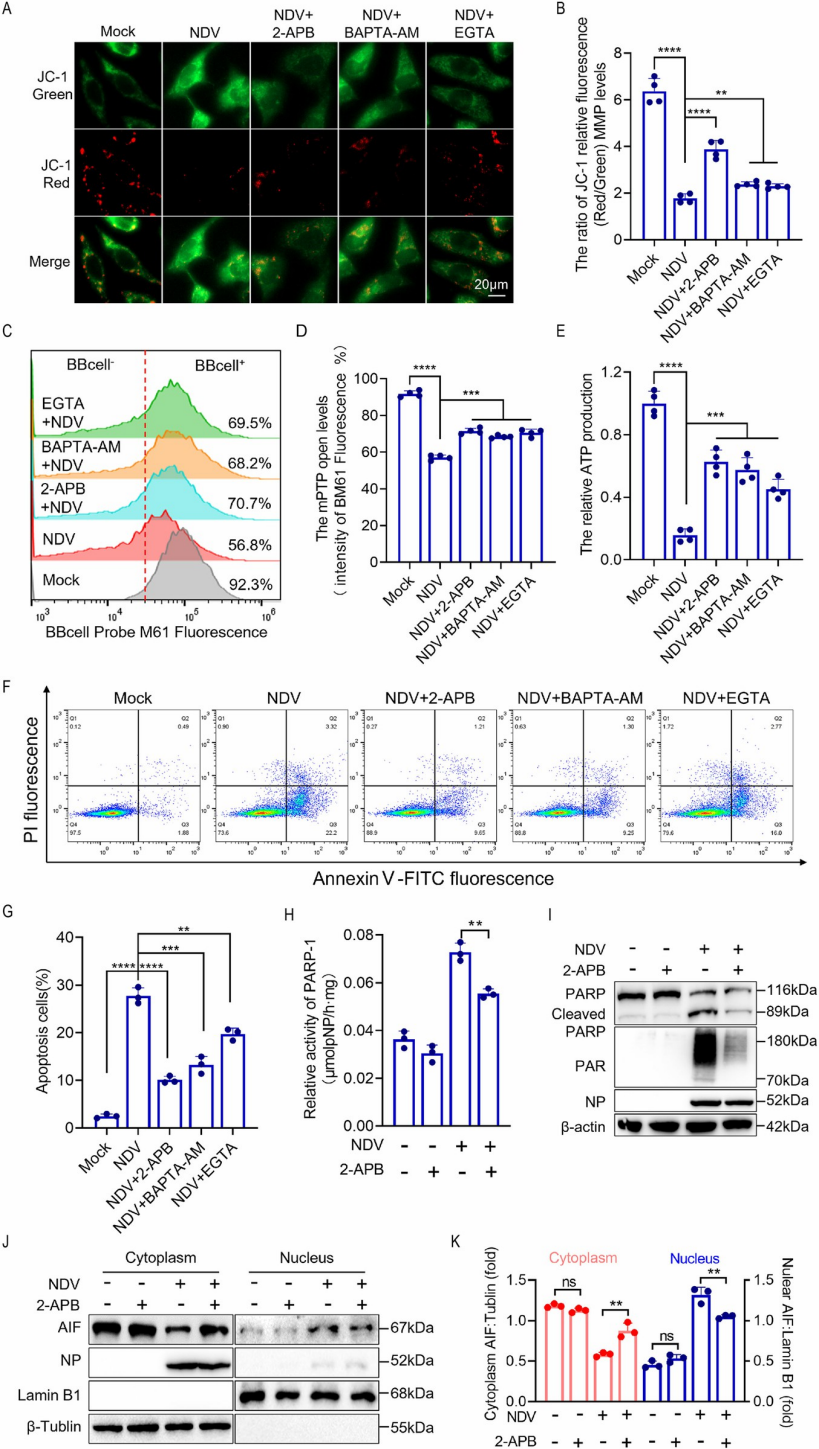
Mitochondrial Ca^2+^ overload induces loss of mitochondrial function and promotes AIF-dependent parthanatos. (A-H) HeLa cells were mock treated, or treated with 2-APB, BAPTA-AM, or EGTA followed by infection with NDV at an MOI of 1 for 18 h. The MMP was assessed using JC-1 staining, with results observed under a fluorescence microscope (A) and quantified (B). The mPTP opening was evaluated through flow cytometry (C) and subsequently quantified (D). (E) ATP production was measured. Apoptosis was detected by flow cytometry (F) and subsequently quantified (G). PARP activity was determined by spectrophotometric (H). (I-K) HeLa cells were treated with or without 2-APB, then mock treated or infected with NDV at an MOI of 1. Cells were harvested at 18 h post-infection. Western blot analyses of the PARP and PAR protein levels (I). Western blot analyses of the levels of AIF amount in cytosol and nucleus. Lamin B1 was used as loading control for nucleus and β-tublin for cytosol (J). Quantification of AIF in cytosol and nucleus (K). The MOI of NDV was set at 1 in all the experiments mentioned above. Each bar represents the mean ± standard deviation; **P < 0.01, ***P < 0.001, ****P < 0.0001 and ns, not significant.

To further investigate the role of NDV-induced Ca^2+^ overload in mitochondrial dysfunction, we evaluated mitochondrial function following the inhibition of Ca^2+^ transfer. As anticipated, treatment with Ca^2+^ inhibitors mitigated NDV-induced dissipation of the MMP (Fig 5A and 5B), mitochondrial membrane permeabilization (Fig 5C and 5D), and ATP production (Fig 5E). Notably, 2-APB demonstrated a more pronounced inhibitory effect on mitochondrial dysfunction induced by NDV infection compared with the other two inhibitors, BAPTA-AM and EGTA. These findings suggest that NDV infection leads to intracellular Ca^2+^ accumulation and mitochondrial Ca^2+^ overload, resulting in structural damage and functional impairment of the mitochondria.

In our subsequent evaluation, we investigated the role of NDV-induced Ca^2+^ overload in the intrinsic mitochondrial pathway of apoptosis. Treatments with 2-APB, BAPTA-AM, and EGTA effectively inhibited apoptosis resulting from NDV infection (Fig 5F and 5G). Notably, 2-APB exhibited a pronounced inhibitory effect on NDV-induced mitochondrial damage and apoptosis, prompting us to select it for further exploration of its regulatory effects on AIF-dependent apoptosis. Treatment with 2-APB significantly inhibited PARP activity (Fig 5H), and Western blot analysis revealed that 2-APB treatment also reduced both PARP cleavage and PAR formation (Fig 5I). Additionally, 2-APB effectively inhibited NDV-induced AIF nuclear translocation (Fig 5J and 5K). We also investigated the impact of 2-APB on virus replication. The results indicated that 2-APB significantly inhibited the expression of NDV NP protein as well as the production of progeny viruses (S5C and S5D Fig). The collective findings indicate that 2-APB inhibits NDV infection-induced mitochondrial structural damage and loss of function by preventing ER Ca^2+^ release. Ultimately, this inhibition leads to a reduction in NDV-induced AIF-dependent parthanatos.

### ROS contributes to NDV-induced AIF-dependent parthanatos

**Fig 6:**
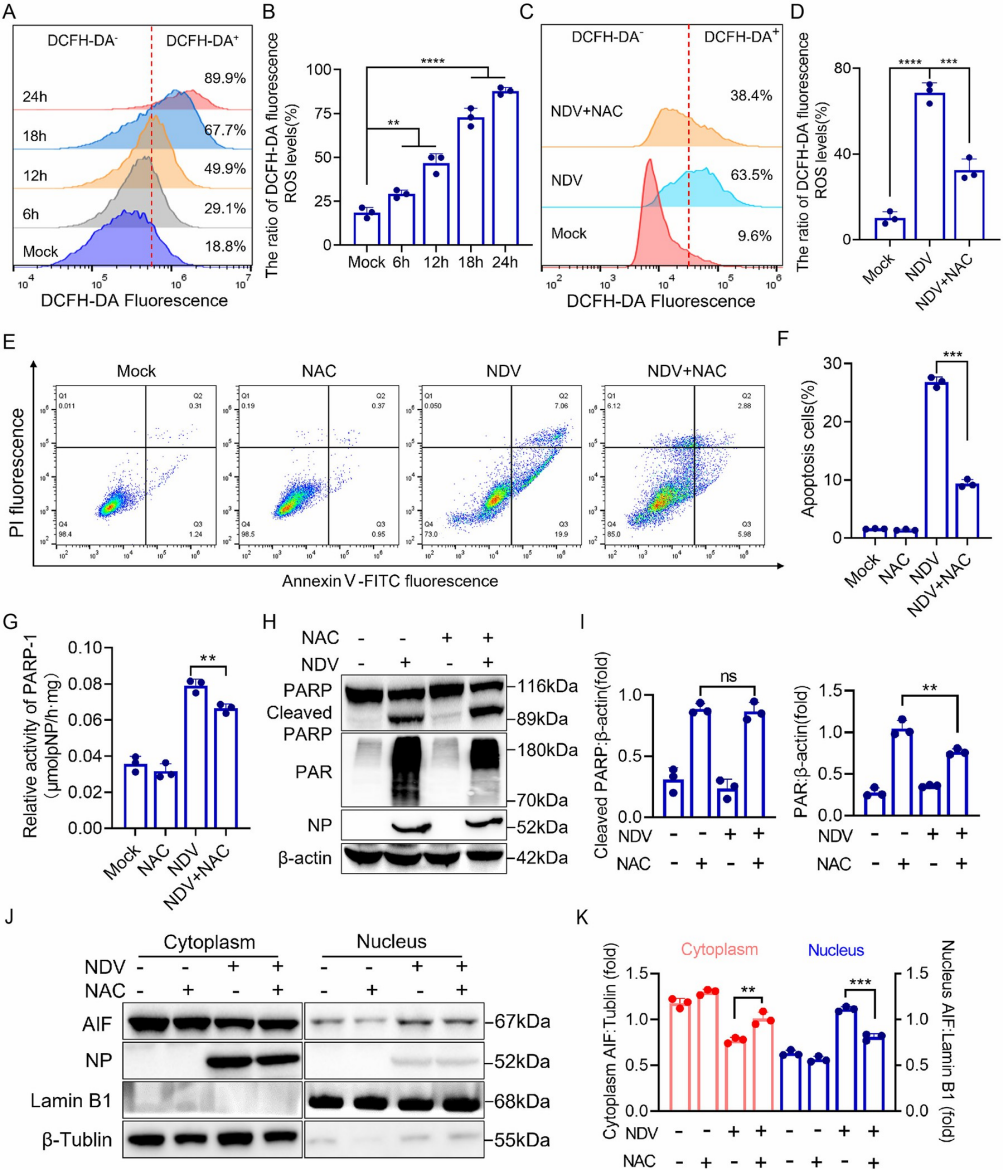
ROS contributes to NDV-induced AIF-dependent parthanatos. (A-B) HeLa cells were mock treated or infected with NDV at 6, 12, 18, and 24 h, ROS production was tested by flow cytometry (A) and subsequently quantified (B). (C-K) HeLa cells were treated with NAC (100 μM) followed by NDV infection for 18 h. ROS production was determined by flow cytometry (C) and subsequently quantified (D). Apoptosis was detected by flow cytometry (E) and quantified (F). PARP activity was determined by spectrophotometric (G). PARP and PAR protein levels determined by western blot (H) and quantification of cleaved PARP and PAR (I). Western blot analyses of the levels of AIF in cytosol and nucleus (J) followed by quantification of AIF in cytosol and nucleus (K). The MOI of NDV was set at 1 in all the experiments mentioned above. Each bar represents the mean ± standard deviation; **P < 0.01, ***P < 0.001, ****P < 0.0001 and ns, not significant.

As a by-product of mitochondrial oxidative phosphorylation, excessive production of ROS leads to mitochondrial damage subsequently triggering mitochondrion-mediated cell death [50,51]. Several studies have demonstrated that ROS contributes to AIF-dependent apoptosis and activates PARP by directly damaging DNA [32,52]. To confirm the role of ROS in NDV-induced apoptosis, we first measured changes in ROS generation following NDV infection using the redox-sensitive fluorescent probe 2′,7′-dichlorofluorescein diacetate (DCFH-DA). ROS production was detected at 6 h after NDV infection and continued to rise as the duration of the infection increased (Fig 6A and 6B). Treatment with the antioxidant compound N-acetyl cysteine (NAC) effectively reversed both NDV-induced ROS production (Fig 6C and 6D) and NDV-induced apoptosis (Fig 6E and 6F). To further investigate the role of ROS in AIF-dependent apoptosis, we assessed the impact of NAC treatment on PARP activity, PAR production, and AIF nuclear translocation. The results revealed that NAC treatment effectively inhibited the increase in PARP activity induced by NDV (Fig 6G). Although NAC did not reduce NDV-induced PARP cleavage, it significantly downregulated total PARP levels, PAR formation (Fig 6H and 6I), and the nuclear translocation of AIF (Fig 6J and 6K). Collectively, these findings suggest that the ROS burst triggered by NDV infection promotes AIF-dependent parthanatos through the activation of PARP.

### ROS promote ER calcium release and mitochondrial dysfunction

**Fig 7:**
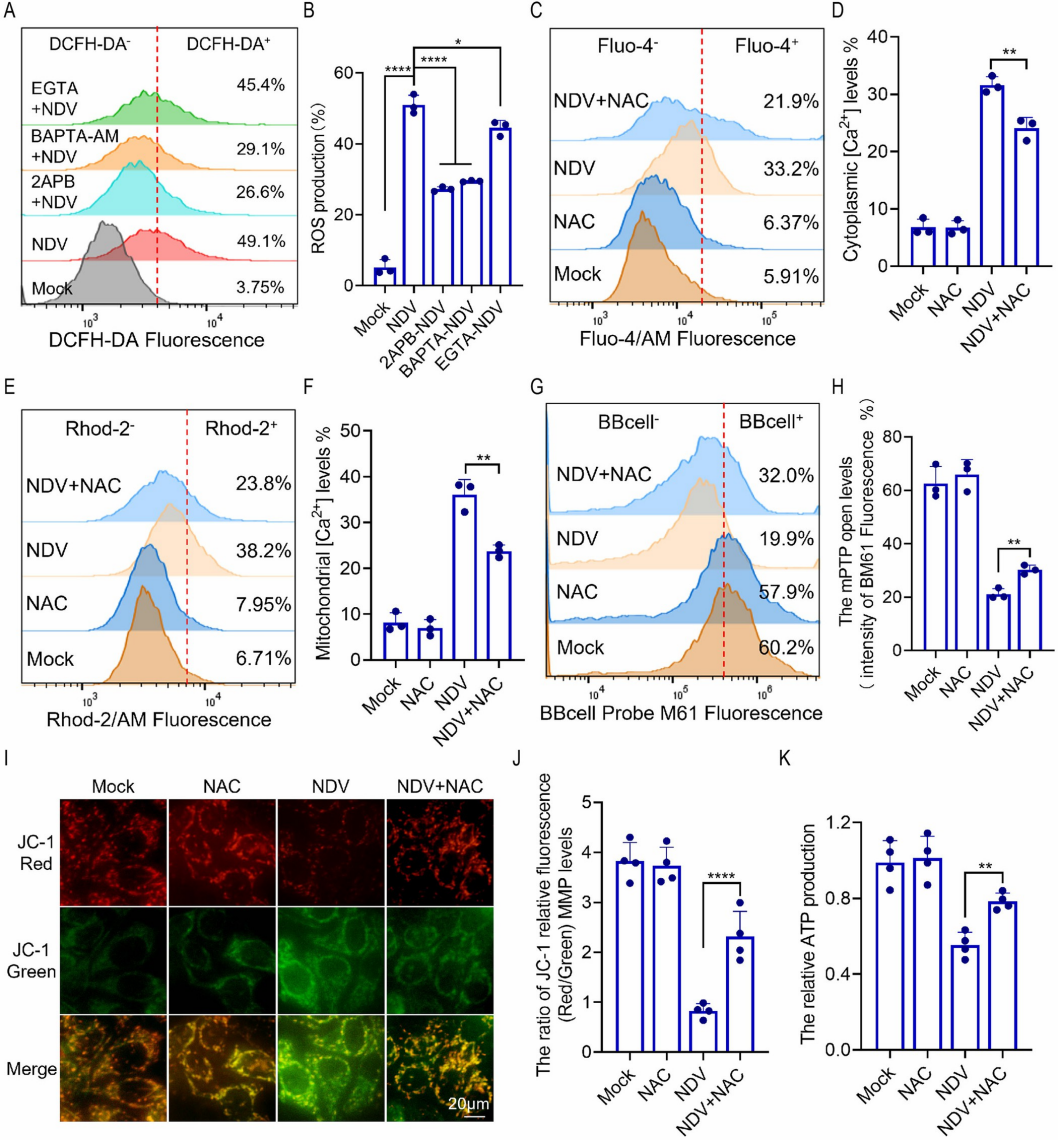
ROS promote ER calcium release and mitochondrial dysfunction and damage. (A-B) HeLa cells were mock treated, or treated with 2-APB, BAPTA-AM, or EGTA followed by NDV infection for 18 h. ROS production was tested by flow cytometry (A) and quantified (B). (C-K) Cells were treated with NAC followed by NDV infection for 18 h. Cytoplasmic Ca^2+^ (C and D) and mitochondria Ca^2+^ (E and F) were detected by flow cytometry and quantified. The mPTP opening detected by flow cytometry (G) and subsequently quantified (H). The MMP was assessed using JC-1 staining, with results observed under a fluorescence microscope (I) and quantified (J). (K) ATP production was measured. The MOI of NDV was set at 1 in all the experiments mentioned above. Each bar represents the mean ± standard deviation; *P < 0.05, **P < 0.01, ****P < 0.0001 and ns, not significant.

NDV infection leads to increased intracellular levels of ROS and Ca^2+^. Both Ca^2+^, recognized as a signaling molecule, and ROS, a byproduct of oxidative stress, are known to promote apoptosis. Consequently, we investigated the potential relationship between ROS and Ca^2+^ during NDV infection. Our findings indicate that NDV-induced mitochondrial Ca^2+^ overload results in mitochondrial damage, prompting us to hypothesize that inhibiting Ca^2+^ elevation could reduce ROS overproduction by mitigating mitochondrial damage. Treatment with 2-APB, BAPTA-AM, or EGTA effectively decreased ROS production, particularly when intracellular Ca^2+^ sources were blocked using 2-APB and BAPTA-AM (Fig 7A and 7B). These results strongly suggest that the increase in intracellular Ca^2+^ contributes to the ROS surge observed during NDV infection. Notably, treatment with NAC also significantly inhibited the accumulation of cytoplasmic and mitochondrial Ca^2+^ in the context of NDV infection (Fig 7C–7F). Furthermore, similar to the effects of 2-APB in inhibiting Ca^2+^ release from the ER, NAC treatment was found to prevent the opening of the mPTP (Fig 7G and 7H), the dissipation of MMP (Fig 7I and 7J), and the reduction of ATP synthesis (Fig 7K) induced by NDV infection. These findings underscore the role of ROS production in the mitochondrial oxidative damage associated with NDV infection. Collectively, our results demonstrate that ROS generated by NDV-induced oxidative stress facilitates the release of Ca^2+^ from the ER, resulting in mitochondrial calcium overload and depolarization, which ultimately leads to mitochondrial ROS accumulation and apoptosis.

## Discussion

**Fig 8:**
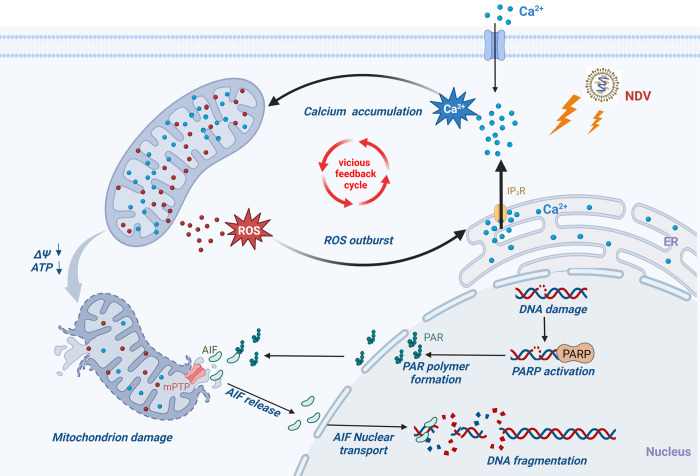
Schematic calcium-mitochondria-ROS signal pathway in NDV-induced AIF-dependent parthanatos. NDV infection induces intracellular calcium accumulation, primarily resulting from the release of calcium from the ER. This overload of mitochondrial calcium uptake leads to mitochondrial depolarization and a reduction in ATP synthesis, which in turn promotes the production of ROS within the mitochondria. These ROS can directly induce parthanatos or target ER calcium channels, facilitating further Ca^2+^ release and establishing a vicious feedback cycle. Additionally, the opening of the mPTP results in the release of AIF from the mitochondria, which is then transported to the nucleus, leading to DNA fragmentation and ultimately triggering parthanatos.

As an acute infectious pathogen and oncolytic agent, NDV specifically targets and kills tumor cells by inducing apoptosis. In this study, we emphasize the critical role of mitochondrial damage in the apoptotic pathway, demonstrating that Ca^2+^ and ROS collaboratively regulate apoptosis induced by AIF in response to NDV. NDV infection leads to significant intracellular accumulation of Ca^2+^, which in turn causes mitochondrial calcium overload, exacerbating mitochondrial stress, depolarization, membrane permeability alterations, and ROS accumulation. These events collectively contribute to mitochondrial damage. Ultimately, the compromised mitochondria release AIF, which translocates to the nucleus to initiate apoptosis. Furthermore, ROS also promotes the increased release of Ca^2+^ from the ER, establishing a detrimental feedback loop that further accelerates the parthanatos process (Fig 8).

Apoptosis is a series of strictly controlled cellular suicide mechanisms governed by complex regulatory pathways. For viruses, apoptosis presents a double-edged sword. On one hand, the host utilizes apoptosis as a self-protective defense mechanism to eliminate infected cells through the interaction of various stages of the apoptotic pathway, thereby limiting viral infection. Conversely, viruses have evolved multiple strategies to evade immune responses by inhibiting apoptosis. Additionally, viruses can deplete host resources and induce cell apoptosis to facilitate viral replication and the release of progeny viruses, promoting further invasion [3]. Indeed, our studies demonstrate that the inhibition of either apoptotic pathway hampers viral replication and the proliferation of progeny viruses. This finding suggests that NDV enhances the shedding and dissemination of its progeny by inducing apoptosis in infected cells, which in turn facilitates the spread of the virus to neighbor cells and accelerates the establishment of acute infection. This study demonstrates that CsA and 2-APB, which function as inhibitors of mPTP opening and mitochondrial calcium overload, respectively, can effectively inhibit NDV-induced apoptosis. However, the inhibitory effects of CsA and 2-APB on viral replication were not observed at high infectious doses. This lack of inhibition may primarily stem from the rapid proliferation of NDV in HeLa cells, resulting in significant cellular damage and death. And at the late stage of viral infection, the proliferative capacity of the virus overcomes the inhibitory effects of the drugs.

In this study, we demonstrated that both caspase knockout and AIF knockout led to a similar reduction in apoptotic cells, suggesting that both types of cell death contribute equally to overall apoptosis. Our previous research has established that NDV infection can induce cell death through multiple pathways, including autophagy, necroptosis, and ferroptosis. It is important and meaningful to identify the primary mechanism of cell death induced by NDV infection. Unfortunately, current detection methods limit our ability to precisely quantify the contribution of each cell death mechanism. It is crucial to recognize that NDV is a potent virus capable of inflicting significant stress damage to cells, thereby activating various regulatory pathways for cell death. The predominant mechanism may vary depending on factors such as cell type, infection time, and virus strain. Furthermore, the interplay between different forms of cell death is intricate; for instance, several soluble mediators play dual roles in both apoptosis and parthanatos by inducing mitochondrial dysfunction and triggering cell death. This highlights the complexity and diversity of the cell death mechanisms triggered by NDV.

Given the high similarity between apoptosis and parthanatos, it is essential to explore the crosstalk between these two processes. Several soluble mediators exhibit dual roles in both apoptosis and parthanatos by inducing mitochondrial dysfunction and triggering cell death. ROS and Ca^2+^ are central to both pathways. In each pathway, ROS bursts and Ca^2+^ overload contributes to mitochondrial permeability, resulting in either Cyt C release for apoptosis or AIF translocation for parthanatos. Cytokines such as tumor necrosis factor-alpha (TNF-α) are well-known for activating the extrinsic apoptotic pathway; however, they can also induce cellular stress, including oxidative stress and ER stress, which lead to mitochondrial dysfunction and AIF-mediated parthanatos [53,54]. Nitric oxide (NO) promotes apoptosis through oxidative stress and caspase activation, but in the context of parthanatos, NO enhances mitochondrial membrane permeability, facilitating AIF release [55–57]. These mediators function as upstream regulators of cellular stress, contributing to both caspase-dependent apoptosis and AIF-dependent parthanatos, albeit through distinct mechanisms of mitochondrial damage and death signaling. Our study provides clear evidence that NDV induces not only apoptosis but also parthanatos. Understanding the shared roles of these pathways offers valuable insights into potential therapeutic targets in NDV-induced cell death mechanisms.

An imbalance in intracellular Ca^2+^ concentration serves as a critical signal during viral infections; however, the specific sources of Ca^2+^ remain largely unexplored. In this study, we demonstrate that NDV infection induces intracellular Ca^2+^ accumulation and mitochondrial overload by facilitating the release of Ca^2+^ from the ER. Similar to Ca^2+^, ROS act as significant activators of cell damage and apoptosis, primarily generated by oxidative stress from the respiratory chain in the inner mitochondrial membrane. When mitochondria are stimulated by external factors, ROS production markedly increases [58]. We demonstrate that mitochondrial calcium overload exacerbates the ROS burst, while ROS production is also critical for the accumulation of Ca^2+^. The substantial buildup of intracellular ROS exacerbates the stress-induced damage to other organelles. Consequently, we propose that ROS facilitates Ca^2+^ release by inducing ER stress, thereby creating a feedback loop of emergency stress involving calcium signaling.

In conclusion, we identified the regulatory mechanisms by which Ca^2+^ and ROS mediate NDV-induced apoptosis dependent on AIF, through the Ca^2+^-mitochondria-ROS signaling pathway. Specifically, NDV induces mitochondrial damage by promoting Ca^2+^ accumulation and excessive ROS production, which facilitates the nuclear translocation of AIF and ultimately triggers AIF-dependent parthanatos. In summary, this study elucidates that NDV utilizes Ca^2+^ and ROS as signaling molecules to induce cell apoptosis and facilitate viral proliferation. These findings contribute to a deeper understanding of the novel mechanisms underlying NDV oncolysis and offer valuable insights and a foundation for future antiviral research.

## Materials and methods

### Cells and viruses

The HeLa, A549, H1299, HepG2, Huh7, HT-29, MCF7 and DF-1 cell lines were obtained from the American Type Culture Collection (ATCC; Manassas, VA, USA). *Casp3*^*-/-*^ HeLa cells were sourced from EdiGene Inc. (Beijing, China) and validated through immunoblotting. *AIF*^*-/-*^ HeLa cells were constructed in our laboratory and also validated by immunoblotting. Cells were cultured in Dulbecco’s modified Eagle’s medium (DMEM; Gibco, Franklin Lakes, NJ, USA) supplemented with 10% fetal bovine serum (Gibco) at 37°C in a 5% CO_2_ atmosphere. The NDV velogenic strain Herts/33, Mukteswar and LaSota were obtained from the China Institute of Veterinary Drug Control in Beijing, China. The NDV GFP-LaSota strains were made and stored in our laboratory The virus was propagated in chicken embryonated eggs and the titers were determined as the median tissue culture infective doses (TCID_50_) on DF-1 cells.

### Reagents and antibodies

The caspase inhibitor Z-VAD-FMK (HY-16658B), the PARP inhibitor 3-AB (HY-12022), the intracellular calcium chelator BAPTA-AM (HY-100545), the extracellular calcium chelator EGTA (HY-D0973), and the mPTP opening inhibitor CsA (HY-B0579) were purchased from MedChemExpress (Monmouth Junction, NJ, USA). The IP_3_R inhibitor 2-APB (T4693) was purchased from Topscience (Shanghai, China). The NAC antioxidant (S1623) was purchased from Selleck Chemicals (Houston, TX, USA). A monoclonal antibody against NDV nucleoprotein (NDV-NP) was prepared in our laboratory. Antibodies against caspase-9 (9504), caspase-8 (4790), caspase-6 (9762), caspase-7 (9492), caspase-3 (14220), PARP (9542), PAR (83732), BAX (41162), OPA1 (80471), MFN1 (14739), MFN2 (11925), and Lamin B1 (13435) were purchased from Cell Signaling Technology (Beverly, MA, USA). Antibody against AIF (ab32516) was purchased from Abcam (Cambridge, UK). Antibody against Cyt C (10993-1-AP) was purchased from Proteintech (Wuhan, China). Horseradish peroxidase conjugated antibodies against β-tubulin (AC021), β-actin (AC006), goat anti-rabbit IgG (H+L) (AS014), and goat anti-mouse IgG (H+L) (AS003) were purchased from ABclonal (Wuhan, China). Alexa Fluor goat anti-rabbit-488 (A11034), Alexa Fluor goat anti-rabbit-594 (A11037), Alexa Fluor goat anti-mouse-488 (A11029), and Alexa Fluor goat anti-mouse-594 (A11005) were obtained from Invitrogen (Carlsbad, CA, USA).

### Flow cytometry analysis of apoptosis

HeLa cells were grown to monolayer in 6-well tissue culture plates, then infected with NDV Herts/33. Apoptosis was detected using the Apoptosis Detection Kit (C1062L, Beyotime, Shanghai, China) according to the manufacturer’s instruction. Briefly, the virus-infected cells were harvested at 18 h post infection (hpi) by centrifugation at 1000 g for 5 min. The cells were then rinsed once with PBS, resuspended in 195 μl Annexin V-FITC binding buffer, followed by incubation with Annexin V and PI at 25°C for 20 min in the dark. The preparations were analyzed by flow cytometry using a flow cytometer (Beckman, California, USA) equipped with FlowJo software.

### Transfection of plasmids or siRNAs

HeLa cells were transfected with plasmids or siRNAs using Lipofectamine 2000 reagent (Thermo Fisher Scientific, Waltham, MA, USA) or PlusTrans transfection reagent (NULEN Biotech, Shanghai, China) according to the manufacturer’s instructions. Briefly, 2 μg plasmid and 4 μl Lipofectamine 2000 (m/v = 1:2), or 5 μl siRNA and 5 μl PlusTrans (v/v = 1:1) were incubated in 0.2 ml Opti-MEM (Gibco) for 5 min, then mixed and incubated at room temperature for 15 min, allowing the formation of lipid–plasmid complex. Finally, the complex was added to 6-well tissue culture plates. At 6 h incubation at 37°C, cells were washed three times with PBS and incubated for an additional 24 h (plasmid transfection) or 36 h (siRNA transfection) before virus infection. The pEGFP-Mito and pDsRed-ER plasmids were prepared in our laboratory. The siRNA targeting endogenous AIF (siAIF 1^#^: 5’-CGGGAAGUCAAAUCAAUUATT-3’; siAIF 2^#^: 5’- GCAUGCUUCUACGAUAUAATT-3’) and Bax (siBax:5’-CUGAUCAGAACCAUCAUGGTT-3’) were purchased from Gene Pharma (Shanghai, China).

### Nucleocytoplasmic separation and mitochondria isolation assays

Nuclear extracts were prepared using NE-PER nuclear and cytoplasmic extraction reagents (78833, Thermo Fisher Scientific) according to the manufacturer’s instructions. Mitochondrial proteins were isolated and extracted using a cell mitochondria isolation kit (C3601; Beyotime) according to the manufacturer’s instructions.

### Western blot

Cells were obtained and lysed in 2× protein loading buffer (20 mM Tris-HCl, 2% SDS, 100 mM dithiothreitol, 20% glycerol, and 0.016% bromophenol blue). The lysates were denatured and the proteins resolved by 10% SDS-PAGE. The proteins were transferred to a nitrocellulose membrane (NC-a101-b105; Whatman, Maidstone, UK). Each membrane was blocked in skim milk for 1 h at room temperature and then incubated with primary antibodies overnight at 4°C, followed by incubation with secondary antibodies for 2 h at room temperature. The protein bands were visualized using the Tanon 4600 Chemiluminescent Imaging System (Bio Tanon, Shanghai, China).

### Indirect immunofluorescence and confocal microscopy

HeLa cells were seeded on cover slips in a 12-well plate for 24 h. The cells were then transfected with plasmids, infected with NDV or incubated with Mito-Tracker (C1049B; Beyotime), fixed with 4% paraformaldehyde for 30 min, permeabilized with 0.5% Triton X-100 for 15 min, and blocked in 3% bovine serum albumin in PBS for 45 min. Cells were incubated with primary antibody at 4°C overnight, followed by incubation with Alexa Fluor conjugated secondary antibody for 1 h at 37°C. Next, the cells were incubated with 0.5 mg/ml 4′,6-diamidino-2-phenylindole (DAPI) for 15 min. Between and after each incubation step, the monolayer of cells were washed three times with a blocking buffer. Finally, cells were washed once with PBS and visualized by confocal microscopy using a model LSM880 confocal microscope (Carl Zeiss, Jena, Germany). Images were analyzed using ImageJ software (NIH, Bethesda, MD, USA).

### Measurement of calcium level

HeLa cells were plated in 6-well plates, pretreated with different reagents, and infected with NDV until the indicated time point. To measure ER calcium, cells were stimulated with 10 μmol/l ionomycin [49]. Cells were then loaded with the fluorescent calcium indicator Fluo-4/AM (5 μM) and Rhod-2/AM (5 μM) for 30 min in the dark to measure intracellular and mitochondrial Ca^2+^ levels, respectively. After being washed twice with PBS, cells were resuspended in 500 ml Hanks’ Balanced Salt Solution (Beyotime) and immediately analyzed by flow cytometry using excitation/emission wavelengths of 488/525 nm for Fluo-4/AM and 549/578 for Rhod-2/AM, or were observed directly by fluorescence microscopy (Olympus, Tokyo, Japan).

### TEM

HeLa cells were seeded in a 10-cm dishes, then infected with NDV. The cells were collected at 18 h post-infection, washed twice with PBS, and fixed with 2.5% glutaraldehyde at 4°C for 30 min. The cell masses were dehydrated in a series of acetone solutions and embedded in epoxy resin (SLCJ5080; Sigma-Aldrich, St. Louis, MO, USA). The ultrathin sections that were cut were observed by TEM using a CM-120 microscope (Philips, Amsterdam, The Netherlands) operating at 80 kV.

### Detection of Caspase and PARP activity

HeLa cells were were cultured to form a monolayer in 6-well cell culture plates, followed by infection with NDV or treatment with reagents. According to the manufacturer’s instructions for the Caspase-6 Activity Assay Kit (E-CK-A386; Elabscience), the Caspase3/7 Activity Assay Kit (E-CK-A383; Elabscience) and the PARP Activity Assay Kit (GMS50116.1; Genmed Scientifics), luminescence measurements of both the standards and samples were obtained using a luminometer.

### Detection of ROS and mPTP activity by flow cytometry

HeLa cells were infected with NDV or pretreated with different reagents for the indicated times. The cells were then incubated at 37°C for 20 min with DMEM containing DCFH-DA (S0033S; Beyotime) at a final concentration of 10 μM. After washing twice with PBS, the cells were analyzed by flow cytometry at excitation/emission wavelengths of 488/525 nm.

mPTP activity was detected with the BBcellProbe M61 probe (BestBio, Shanghai, China). After various treatments, the cells were harvested with trypsin, then suspended in HBSS. Each cell sample was treated with 5 μl BBcellProbe M61 probe (200 μM) for 5 min and quencher for 15 min at 37°C. The cells were then washed three times with PBS, resuspended in HBSS and analyzed by flow cytometry at excitation/emission wavelengths of 488/525 nm. Opening of mPTP was evident by decreased fluorescence.

### MMP assay

MMP was detected with JC-1 (C2006; Beyotime). HeLa cells were seeded in 6-well cell culture plates. After various treatments or infection with NDV, the cells were loaded with JC-1 for 20 min at 37°C in the dark and then washed twice with PBS and observed by fluorescence microscopy. Detection of JC-1 monomer and polymer was based on the fluorescein isothiocyanate and Cy3 setting, respectively. Decreased MMP was evident by increased green fluorescence.

### Measurement of ATP levels

An ATP Assay Kit (S0026; Beyotime) was used to test mitochondrial ATP production rate. HeLa cells were grown to monolayer in 6-well cell culture plates, then infected with NDV or pretreated with reagents. According to the manufacturer’s instruction, a luminometer was used to measure the luminescence rate of the standard and samples. The standard curve was drawn to calculate the concentration of ATP in the sample.

### Statistical analysis

The data are expressed as mean ± standard deviation (SD) of three independent experiments and analyzed statistically by Graphpad Prism8 software (GraphPad, USA). Significant differences among groups were determined with one-way analysis of variance followed by Tukey tests. P-values < 0.05 were considered statistically significant.

## Supporting information

S1 Fig NDV induces apoptosis in tumor cells. (A) Apoptosis was evaluated using flow cytometry at 18 h post-infection with an MOI of 1 for NDV or a mock infection in different tumor cells. (B) HeLa cells were infected with different strains of NDV at an MOI of 1 for 18h, and cell apoptosis was detected by flow cytometry. (C) HeLa Cells were mock treated or infected with NDV at 6, 12, 18, and 24 h, Caspase3/7 and Caspase-6 activity were determined by spectrophotometric. (D and E) HeLa cells were infected with NDV for 18 h or were mock infected. The cells were subjected to immunostaining. Mitochondria were labeled with Mito-Tracker (red), Cyt C (D) and BAX (E) with antibody (green), and cell nuclei with DAPI (blue), Scale bars: 20 μm. Statistical co-location analysis data are displayed on the right. Each bar represents the mean ± standard deviation; *P < 0.05, **P < 0.01, ***P < 0.001, ****P < 0.0001 and ns, not significant.
(TIF)

S2 Fig Knockdown of AIF inhibited NDV-induced apoptosis. (A) Apoptosis was detected by flow cytometry at 18 h post-infection with an MOI of 5 for NDV or a mock infection of WT and Casp3-/- cells. (B) Western blot analyses of the levels of PARP, AIF, and caspase-3 at 18 h post-infection with an MOI of 5 for NDV or mock infection. (C) Transfection with siRNA targeting AIF and detection of the knockdown level of AIF by western blot. (D) Casp3-/- cells were transfected with siRNA targeting AIF. The cells were infected with NDV at 24 h post-transfection, apoptosis was detected by flow cytometry at 18 h post NDV infection or mock infection. (E) The apoptosis rates of cells at different groups.Each bar represents the mean ± standard deviation; ***P < 0.001 and ns, not significant.
(TIF)

S3 Fig NDV infection induces apoptosis by activating PARP pathway. (A)Casp3-/- cells were infected with NDV at an MOI of 1 for 18h, western blot analyses of the AIF amount in cytosol and nucleus. (B) Quantification of AIF in cytosol and nucleus. HeLa Cells were mock treated, or pretreated with 3-AB followed by NDV infection for 18 h. (C) Apoptosis detected by flow cytometry. (D) Quantification of apoptosis. (E) PARP and PAR protein levels determined by western blot. (F) HeLa cells were transfected with siRNA targeting Bax, the cells were infected with NDV at 24 h post-transfection, western blot analyses of the levels of AIF in cytosol and nucleus at 18 h post NDV infection or mock infection. (G) Quantification of AIF in nucleus. Each bar represents the mean ± standard deviation; *P < 0.05, **P < 0.01, ***P < 0.001, ****P < 0.0001 and ns, not significant.
(TIF)

S4 Fig NDV induces mitochondrial structural damage and dysfunction. (A) The degree of mPTP opening was detected by flow cytometry analysis of cells infected with NDV. The peak shift to the left indicates mPTP opening. (B) Quantification of mPTP activity. (C) Electron microscopy observation. Images revealed the mitochondrial ultrastructure of HeLa cells at 18 h post NDV infection or mock infection. The red arrow indicates swollen mitochondria and blurred boundaries of mitochondria. (D) Confocal microscopy images of mitochondrial morphology and fragmentation. Mitochondria, NDV-NP and cell nuclei were labeled with Mito-Tracker (red), anti-NP antibody (green), and DAPI (blue) respectively. (E) OPA1, MFN1, and MFN2 protein levels were determined by western blot using β-actin as the loading control and NP as a marker for virus infection. (F) Quantification of OPA1, MFN1, and MFN2. (G) ATP production of mock treated cells and NDV infected cells. (H) MMP was detected using JC-1 stained samples by fluorescence microscopy with CCCP as a positive control. (I) Quantification of MMP. Each bar represents the mean ± standard deviation; *P < 0.05, **P < 0.01, ***P < 0.001, ****P < 0.0001 and ns, not significant.
(TIF)

S5 Fig CsA and 2-APB inhibited NDV replication and proliferation. (A and B) HeLa cells were treated with or without CsA (10 μM), then mock treated or infected with NDV at an MOI of 0.1 or 1. Cells were harvested at 18 h post-infection. The amounts of viral protein (A) were assessed in the cell lysates, while cell culture supernatants were subjected to the viral titer assay (B). (C and D) HeLa cells were treated with or without 2-APB (100 μM), then mock treated or infected with NDV at an MOI of 0.1 or 1. Cells were harvested at 18 h post-infection. The amounts of viral protein (C) were assessed in the cell lysates, while cell culture supernatants were subjected to the viral titer assay (D). Each bar represents the mean ± standard deviation; *P < 0.05 and ns, not significant.
(TIF)
