# Supplementary figures and images for: Newcastle disease virus infection induces parthanatos in tumor cells via calcium waves
Source: PLoS Pathog. 2024 Dec 2;20(12):e1012737. doi: 10.1371/journal.ppat.1012737 (PMC11637436; doi:10.1371/journal.ppat.1012737)

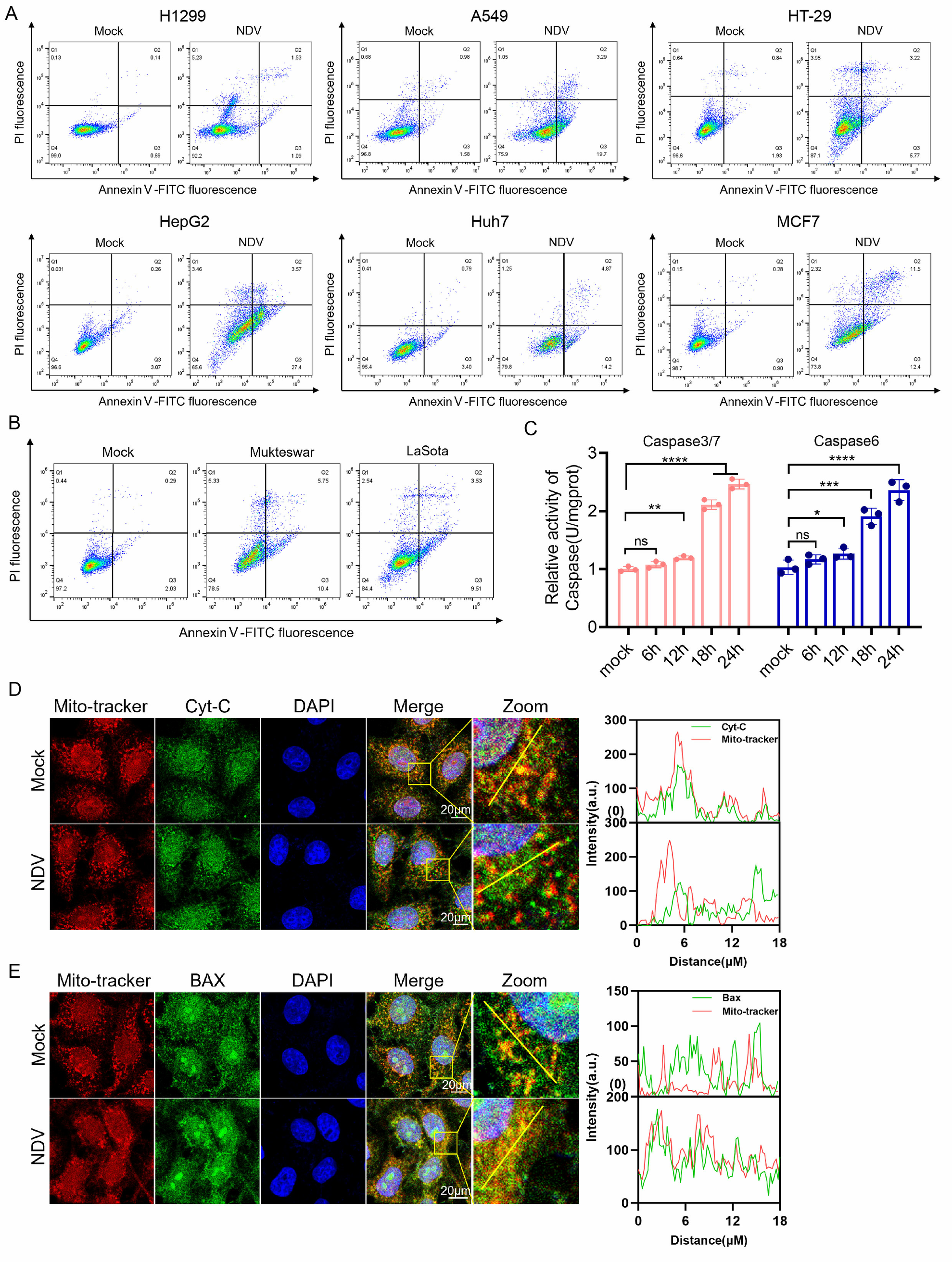

Supplement: S1 Fig — (A) Apoptosis was evaluated using flow cytometry at 18 h post-infection with an MOI of 1 for NDV or a mock infection in different tumor cells. (B) HeLa cells were infected with different strains of NDV at an MOI of 1 for 18h, and cell apoptosis was detected by flow cytometry. (C) HeLa Cells were mock treated or infected with NDV at 6, 12, 18, and 24 h, Caspase3/7 and Caspase-6 activity were determined by spectrophotometric. (D and E) HeLa cells were infected with NDV for 18 h or were mock infected. The cells were subjected to immunostaining. Mitochondria were labeled with Mito-Tracker (red), Cyt C (D) and BAX (E) with antibody (green), and cell nuclei with DAPI (blue), Scale bars: 20 μm. Statistical co-location analysis data are displayed on the right. Each bar represents the mean ± standard deviation; *P < 0.05, **P < 0.01, ***P < 0.001, ****P < 0.0001 and ns, not significant. (TIF) [file ppat.1012737.s001.tif]

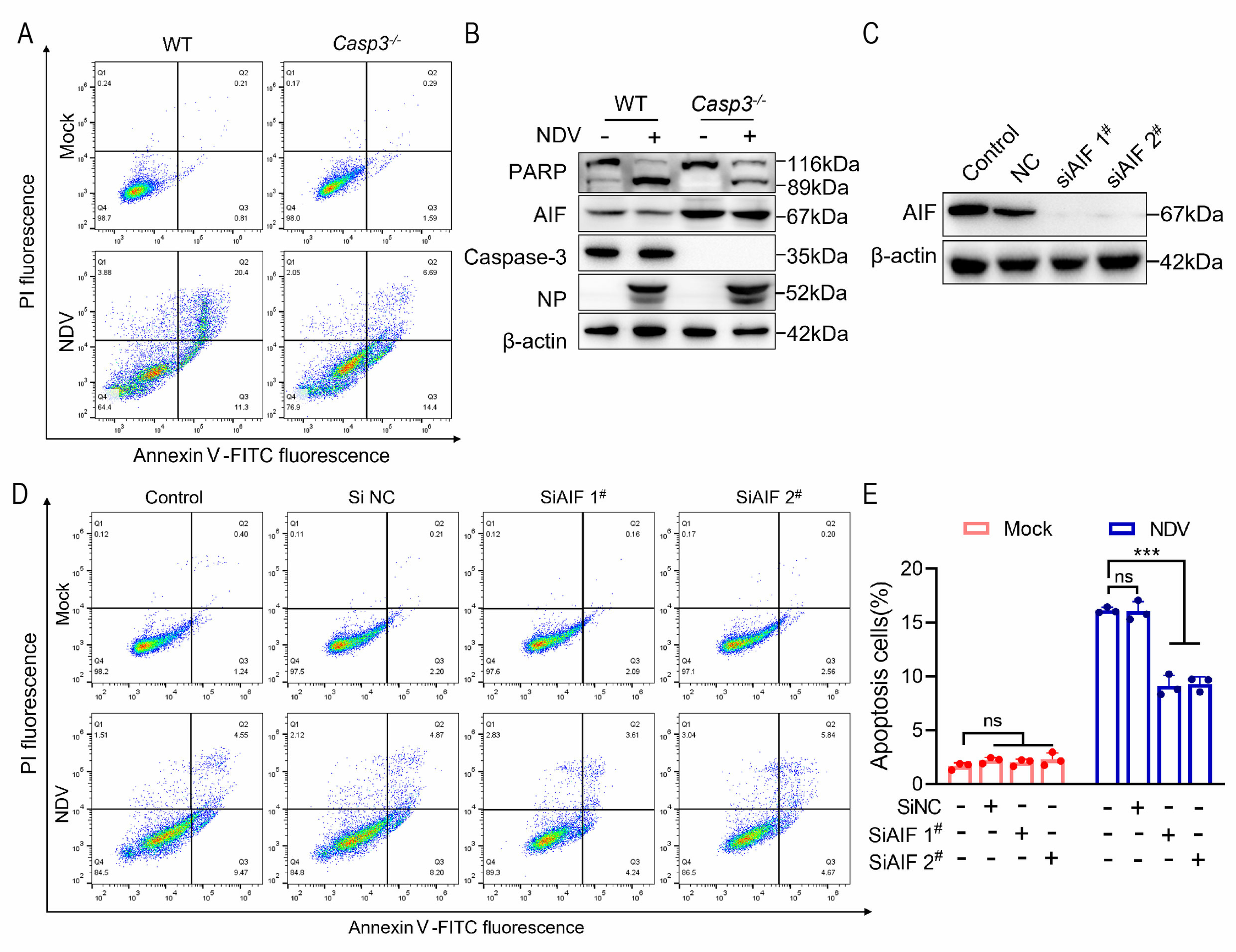

Supplement: S2 Fig — (A) Apoptosis was detected by flow cytometry at 18 h post-infection with an MOI of 5 for NDV or a mock infection of WT and Casp3-/- cells. (B) Western blot analyses of the levels of PARP, AIF, and caspase-3 at 18 h post-infection with an MOI of 5 for NDV or mock infection. (C) Transfection with siRNA targeting AIF and detection of the knockdown level of AIF by western blot. (D) Casp3-/- cells were transfected with siRNA targeting AIF. The cells were infected with NDV at 24 h post-transfection, apoptosis was detected by flow cytometry at 18 h post NDV infection or mock infection. (E) The apoptosis rates of cells at different groups.Each bar represents the mean ± standard deviation; ***P < 0.001 and ns, not significant. (TIF) [file ppat.1012737.s002.tif]

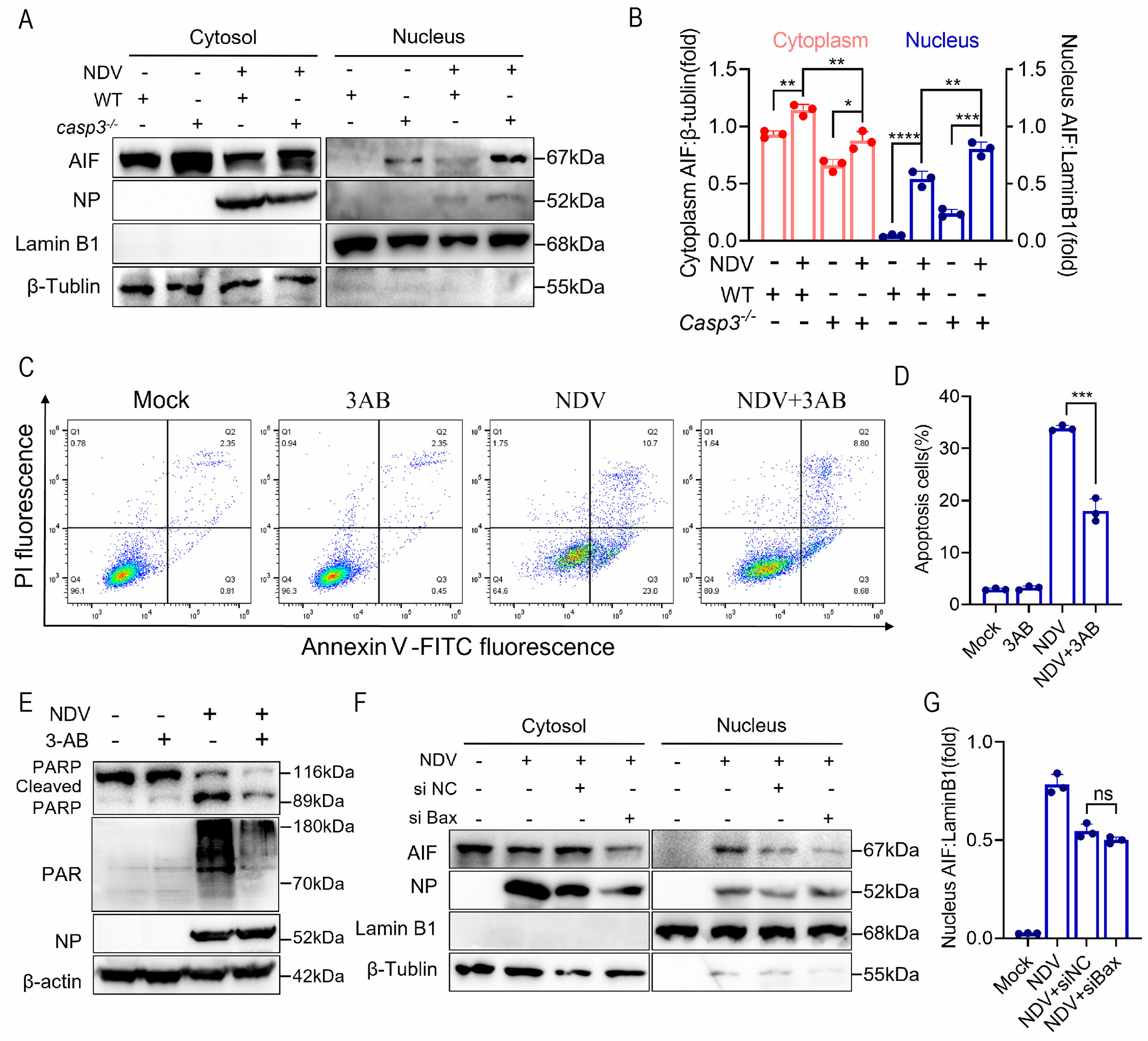

Supplement: S3 Fig — (A)Casp3-/- cells were infected with NDV at an MOI of 1 for 18h, western blot analyses of the AIF amount in cytosol and nucleus. (B) Quantification of AIF in cytosol and nucleus. HeLa Cells were mock treated, or pretreated with 3-AB followed by NDV infection for 18 h. (C) Apoptosis detected by flow cytometry. (D) Quantification of apoptosis. (E) PARP and PAR protein levels determined by western blot. (F) HeLa cells were transfected with siRNA targeting Bax, the cells were infected with NDV at 24 h post-transfection, western blot analyses of the levels of AIF in cytosol and nucleus at 18 h post NDV infection or mock infection. (G) Quantification of AIF in nucleus. Each bar represents the mean ± standard deviation; *P < 0.05, **P < 0.01, ***P < 0.001, ****P < 0.0001 and ns, not significant. (TIF) [file ppat.1012737.s003.tif]

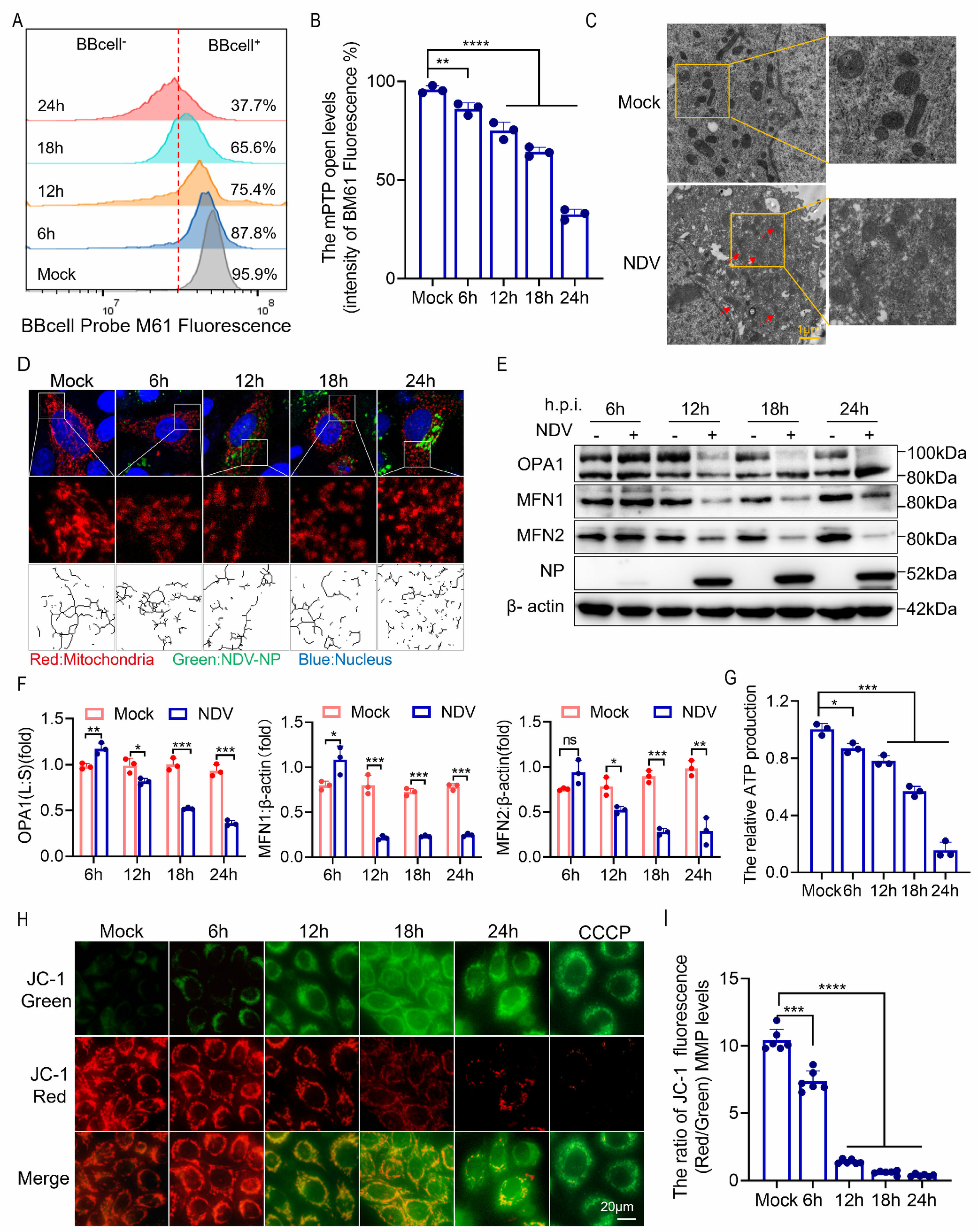

Supplement: S4 Fig — (A) The degree of mPTP opening was detected by flow cytometry analysis of cells infected with NDV. The peak shift to the left indicates mPTP opening. (B) Quantification of mPTP activity. (C) Electron microscopy observation. Images revealed the mitochondrial ultrastructure of HeLa cells at 18 h post NDV infection or mock infection. The red arrow indicates swollen mitochondria and blurred boundaries of mitochondria. (D) Confocal microscopy images of mitochondrial morphology and fragmentation. Mitochondria, NDV-NP and cell nuclei were labeled with Mito-Tracker (red), anti-NP antibody (green), and DAPI (blue) respectively. (E) OPA1, MFN1, and MFN2 protein levels were determined by western blot using β-actin as the loading control and NP as a marker for virus infection. (F) Quantification of OPA1, MFN1, and MFN2. (G) ATP production of mock treated cells and NDV infected cells. (H) MMP was detected using JC-1 stained samples by fluorescence microscopy with CCCP as a positive control. (I) Quantification of MMP. Each bar represents the mean ± standard deviation; *P < 0.05, **P < 0.01, ***P < 0.001, ****P < 0.0001 and ns, not significant. (TIF) [file ppat.1012737.s004.tif]

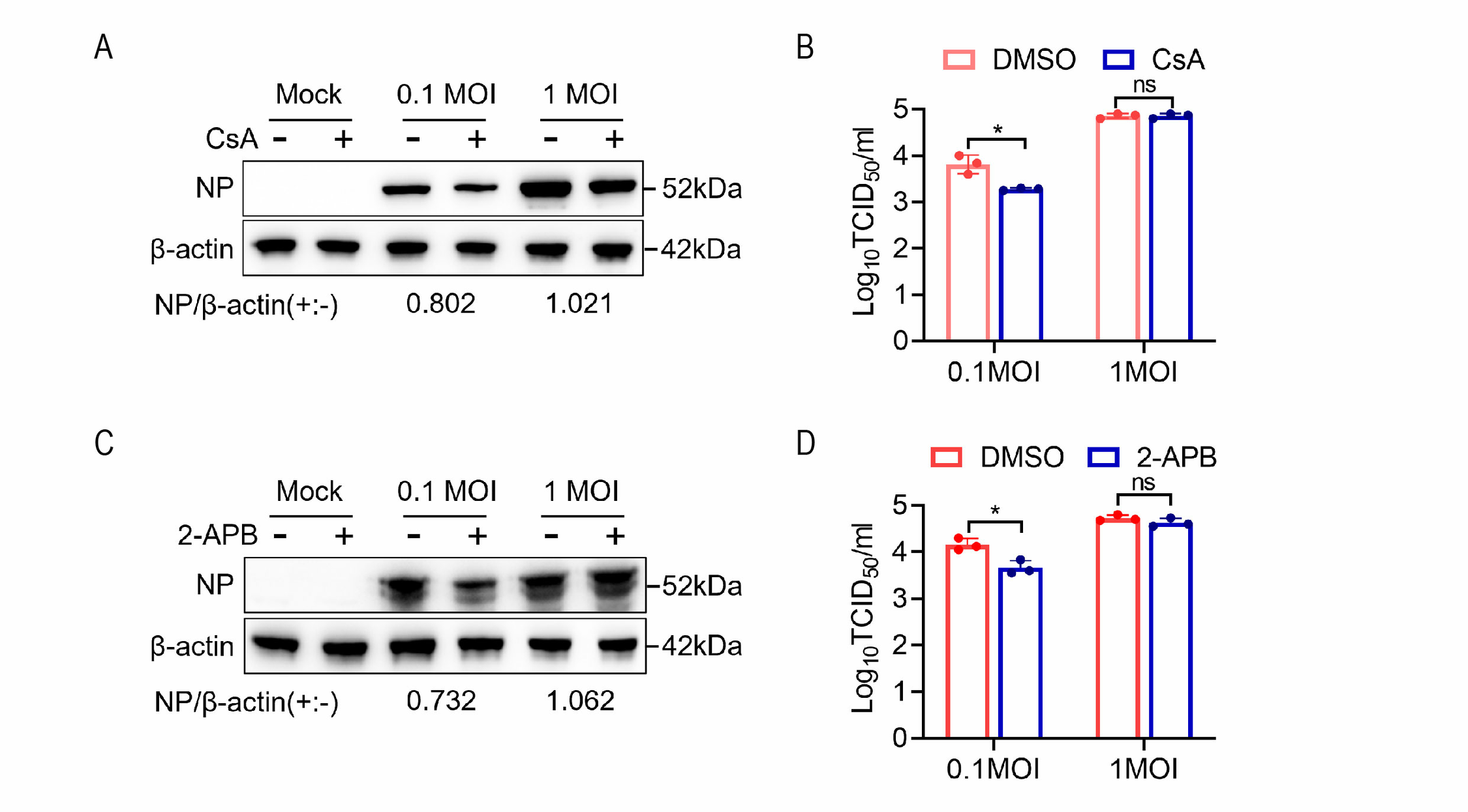

Supplement: S5 Fig — (A and B) HeLa cells were treated with or without CsA (10 μM), then mock treated or infected with NDV at an MOI of 0.1 or 1. Cells were harvested at 18 h post-infection. The amounts of viral protein (A) were assessed in the cell lysates, while cell culture supernatants were subjected to the viral titer assay (B). (C and D) HeLa cells were treated with or without 2-APB (100 μM), then mock treated or infected with NDV at an MOI of 0.1 or 1. Cells were harvested at 18 h post-infection. The amounts of viral protein (C) were assessed in the cell lysates, while cell culture supernatants were subjected to the viral titer assay (D). Each bar represents the mean ± standard deviation; *P < 0.05 and ns, not significant. (TIF) [file ppat.1012737.s005.tif]
